# Intolerance of Uncertainty, Cognitive Avoidance, Positive Beliefs About Worry and Negative Problem Orientation: Relevance to Anxiety Disorders, OCD and Depression in Youth

**DOI:** 10.1002/cpp.70184

**Published:** 2025-11-27

**Authors:** Matti Cervin, Per Andrén, Sean Perrin

**Affiliations:** ^1^ Unit for Child and Adolescent Psychiatry, Department of Clinical Sciences, Lund Lund University Lund Sweden; ^2^ Department of Child and Adolescent Psychiatry Skåne University Hospital Lund Sweden; ^3^ Department of Psychology Lund University Lund Sweden

**Keywords:** adolescents, anxiety, children, cognitive avoidance, intolerance of uncertainty, negative problem orientation, OCD, positive beliefs about worry

## Abstract

Intolerance of uncertainty (IU), cognitive avoidance (CA), positive beliefs about worry (PBW) and a tendency to view everyday problems as *threats*, termed negative problem orientation (NPO), are cognitive vulnerabilities associated with symptoms of anxiety and depression in adults, with fewer studies examining all four vulnerabilities in youth. In this study, validated measures of IU, CA, PBW and NPO were administered to clinically referred youth with a principal diagnosis of obsessive‐compulsive disorder (OCD, *n* = 86), anxiety disorders (*n* = 80) or major depression (*n* = 18) and to non‐clinical peers (*n* = 46). Group differences and the contribution of each vulnerability to internalizing symptom domains were examined. The OCD and anxiety groups did not differ significantly from each other on any vulnerability but had higher scores than non‐clinical peers on all vulnerabilities except PBW. Alongside age and sex, IU, CA, PBW and NPO accounted for 52% of the variance in generalized anxiety symptoms, 51% in depression, 48% in panic, 31% in obsessions/compulsions, 29% in separation anxiety and 18% in social anxiety. Structural modelling revealed that IU was associated with all anxiety symptom domains and that NPO was most strongly associated with depression. These findings suggest that IU, CA, PBW and NPO are linked to various internalizing symptom domains in youth and that IU and NPO act as transdiagnostic vulnerabilities and may be important treatment targets.

## Summary

- IU, CA, PBW and NPO are cognitive vulnerabilities of potential relevance to internalizing disorders in youth
- We examined these vulnerabilities in clinically referred youth with internalizing disorders and in non‐clinical peers
- The clinical groups had more difficulties with IU, CA and NPO than non‐clinical peers
- IU, CA, PBW and NPO were most clearly linked to generalized anxiety, depression and panic
- IU and NPO may act as transdiagnostic vulnerability factors in youth and may represent promising treatment targets

## 1 Introduction

Psychiatric disorders are the leading cause of disability in children and adolescents (hereinafter, youth; ~7–17 years), with internalizing disorders (e.g., anxiety, obsessive‐compulsive and depressive disorders) being most common (Kieling et al. 2024). The first‐line treatment for these disorders is cognitive‐behavioural therapy (CBT). The psychological models that underpin CBT focus on potentially modifiable risk and maintaining factors, including (1) cognitive vulnerabilities (e.g., negative schema/beliefs, attentional biases and negatively biased interpretations of ‘threats’ and one's own coping skills to deal with these threats); (2) having limited or dysfunctional coping skills for dealing with perceived threats and everyday stressors (e.g., behavioural and cognitive avoidance, compulsive rituals, and reassurance‐seeking); and (3) psychosocial factors (e.g., parenting and peer influences, exposure to stressors/punishing experiences) (Benjamin et al. 2011).

In an attempt to better explain the development and maintenance of excessive worry and generalized anxiety disorder (GAD) in adults, Dugas, Freeston and colleagues (Dugas et al. 1998; Freeston et al. 1994) specified four cognitive vulnerabilities in what is often referred to as the Laval model: (1) *Intolerance of Uncertainty* (IU), a negative trait‐like characteristic that results from catastrophic beliefs about uncertainty; (2) *Cognitive Avoidance* (CA), involving thought suppression and substitution, distraction, avoidance of threatening stimuli and the transformation of images into thoughts; (3) *Positive and Negative Beliefs about Worry* (PBW, NBW), referring to beliefs that worries may be dangerous and difficult to control or alternatively that worries may prevent bad things from happening, facilitate problem solving or protect against negative emotions; and (4) *Negative Problem Orientation* (NPO), a cognitive‐emotional tendency to perceive problems as threats, to doubt in one's ability to solve problems and pessimism about the outcome of problem‐solving attempts. The definition of IU has changed somewhat over time but is now generally accepted to refer to a dispositional incapacity to endure the aversive responses triggered by the perceived absence of salient information and sustained by the associated perception of uncertainty (Carleton et al. 2016). Within the Laval model, IU is argued to act as a higher‐order vulnerability factor for excessive worrying and anxiety because it predisposes individuals to use CA and to experience NPO and PBW and NBW, all of which together increase the risk of bouts of excessive worry, particularly in situations involving uncertainty, which in turn contributes to overall levels of anxiety (Dugas et al. 1998).

Although originally formulated to explain GAD, subsequent research has examined each vulnerability in relation to a broader range of disorders, suggesting they may function as transdiagnostic processes. Evidence is strongest for IU, with systematic reviews and meta‐analyses finding moderate to strong associations with anxiety, obsessive‐compulsive and related disorders, depression, PTSD and eating disorders in adults (Brown et al. 2017; Gentes and Ruscio 2011; Gu et al. 2020; McEvoy et al. 2019) and moderate to large associations with worry and anxiety in youth, including those with autism (Jenkinson et al. 2020; Osmanağaoğlu et al. 2018). Individual studies also link IU to the severity of depressive, PTSD and chronic pain symptoms in youth (Fialko et al. 2012; Perrin et al. 2019; Rafieian et al. 2024; Raymond et al. 2022; Soltani et al. 2022).

The definition of CA in the Laval model includes a range of cognitive processes identified in other cognitive models of anxiety, which are often grouped with non‐cognitive strategies in studies of anxiety and depression under broader constructs such as coping, emotion regulation, experiential avoidance and psychological flexibility. Meta‐analyses with adults find moderate to large associations between the severity of symptoms of various disorders and the use of avoidance strategies broadly, including thought suppression and distraction (Akbari et al. 2022; Aldao et al. 2010; Angelakis and Pseftogianni 2021; Haywood et al. 2023; Miethe et al. 2023; Prefit et al. 2019). In youth, findings are more limited: Thought suppression has small associations with anxiety and depression (Schäfer et al. 2017), while psychological inflexibility (involving experiential avoidance) shows larger associations with anxiety (Lønfeldt et al. 2017).

In meta‐analyses with adults, PBW and NBW and related metacognitive beliefs have been found to be moderately to strongly associated with anxiety, and moderately associated with OCD, depression, psychosis and somatic illness (Cotter et al. 2017; Keen et al. 2022; Olatunji et al. 2010). Fewer studies have been carried out with youth, with meta‐analyses reporting weaker and less consistent associations, except for moderate associations between PBW and GAD (Köcher et al. 2021; Lønfeldt et al. 2017; Thingbak et al. 2024).

There is overlap between NPO as defined under the Laval model and D'Zurilla and Nezu's problem‐solving model (D'Zurilla and Nezu 2010), which adds emotional components (e.g., frustration intolerance). Meta‐analytic and individual studies using either definition in adults show moderate associations with worry and stronger associations with GAD and OCD than with depression (Clarke et al. 2017; Dupuy and Ladouceur 2008; Fergus and Wu 2010; Ladouceur et al. 2000). In youth, higher NPO and low problem‐solving confidence are linked to greater severity of fear, worry, anxiety, depression and hostility (Ciarrochi et al. 2009; Donovan et al. 2017; Fialko et al. 2012; Hearn et al. 2017; Kertz and Woodruff‐Borden 2013; Laugesen et al. 2003; Parkinson and Creswell 2011; Wilson and Hughes 2011).

Together, these findings suggest that IU, CA, PBW and NPO may act as important cognitive vulnerabilities linked to anxiety, OCD and depression in both adults and youth, yet most research has examined them separately, with inconsistent terminology and limited focus on youth. Because these vulnerabilities are interrelated and likely reflect a common cognitive‐affective architecture, joint modelling is needed to separate shared (transdiagnostic) from unique effects and to reduce omitted‐variable bias (i.e., bias arising from leaving relevant predictors out of the model). In research with youth, it is also important to consider age, as developmental stage and pubertal timing are likely tied to each vulnerability. Sex/gender differences are also plausible, though their role in clinical samples remains uncertain. To address current research gaps on cognitive vulnerabilities and internalizing disorders in youth, the present study examined these vulnerabilities in a large clinical youth sample to test whether groups with different internalizing disorders differ from each other and from non‐clinical peers and to assess the extent to which the four Laval model factors account for variation in symptom severity across key internalizing symptom domains. This study builds on prior work validating the Laval model in non‐referred youth (Fialko et al. 2012) and through CBT trials targeting IU, CA, NPO and PBW in youth with GAD and comorbid conditions (Perrin et al. 2019; Wahlund et al. 2022; Wahlund et al. 2020). However, given the limited prior work jointly modelling these vulnerabilities in youth, the present analyses were conducted as exploratory and hypothesis‐generating rather than confirmatory and were not preregistered.

## 2 Methods

### 2.1 Participants

We included 184 clinical and 46 non‐clinical youth aged 7–17 years. Clinical participants were consecutively referred to a specialized child and adolescent mental health clinic in Lund, Sweden. Of the 184 included clinical participants, 86 had a principal diagnosis of OCD, 80 had a principal anxiety disorder and 18 had a current principal diagnosis of major depression. Within the anxiety group, 30 had GAD as their principal diagnosis (38%), 27 had social anxiety disorder (34%), 9 had panic disorder (11%), 9 had specific phobia (11%) and 5 had separation anxiety disorder (6%). Non‐clinical participants were recruited from local schools. Sociodemographic and clinical characteristics of the groups are presented in Table 1 alongside scores on the measures used in the study.

**TABLE 1:** Sociodemographic, clinical and measurement information.

	OCD *n* = 86	Anxiety disorders *n* = 80	Depression *n* = 18	Non‐clinical *n* = 46
Sociodemographic information				
Age, *M* (SD)	13.76 (2.52)	14.78 (2.94)	15.13 (2.33)	14.32 (3.04)
Female, *n* (%)	54 (63%)	66 (83%)	15 (83%)	31 (67%)
Living with both parents, *n* (%)^a^	56 (65%)	44 (63%)	5 (56%)	27 (63%)
Mother with university education, *n* (%)^b^	67 (79%)	39 (59%)	3 (60%)	34 (87%)
Father with university education, *n* (%)^b^	43 (51%)	38 (58%)	4 (67%)	29 (78%)
Diagnostic information				
Proportion with major depression, *n* (%)	10 (12%)	24 (31%)	18 (100%)	0 (0%)
Proportion with anxiety disorder, *n* (%)	47 (55%)	80 (100%)	4 (36%)	0 (0%)
Proportion with OCD, *n* (%)	86 (100%)	0 (0%)	0 (0%)	0 (0%)
Proportion with neurodev. dis., *n* (%)	19 (22%)	11 (14%)	2 (15%)	0 (0%)
Proportion with GAD, *n* (%)	30 (35%)	41 (53%)	1 (9%)	0 (0%)
Cognitive measures				
Intolerance of uncertainty (5–25), *M* (SD)	11.28 (5.20)	13.28 (4.88)	11.72 (4.39)	8.18 (3.05)
Positive beliefs about worry (5–25), *M* (SD)	8.85 (3.18)	8.59 (3.37)	8.28 (2.63)	8.30 (2.83)
Cognitive avoidance (5–25), *M* (SD)	15.90 (5.50)	15.89 (4.43)	17.00 (3.71)	12.26 (5.36)
Negative problem orientation (5–25), *M* (SD)	12.78 (5.96)	14.71 (5.31)	17.61 (4.67)	8.17 (3.65)
Symptom measures				
Depression, CDI‐S (0–20), *M* (SD)	5.76 (4.45)	7.80 (4.60)	9.61 (3.76)	2.11 (2.40)
Social anxiety, SCARED‐R (0–14), *M* (SD)	5.77 (3.87)	8.09 (4.29)	7.72 (4.91)	4.00 (3.43)
Panic anxiety, SCARED‐R (0–26), *M* (SD)	7.71 (6.04)	11.12 (5.87)	10.22 (4.36)	3.46 (3.18)
Generalized anxiety, SCARED‐R (0–18), *M* (SD)	9.36 (4.88)	11.67 (4.63)	11.94 (4.53)	5.59 (4.78)
Separation anxiety, SCARED‐R (0–16), *M* (SD)	5.40 (3.57)	5.60 (3.42)	4.50 (4.08)	2.63 (2.76)
OCD, OCI‐CV (0–36), *M* (SD)	16.95 (6.71)	10.98 (5.76)	11.18 (4.67)	6.69 (5.02)

Abbreviations: GAD = generalized anxiety disorder; Neurodev. dis. = neurodevelopmental disorders.

^a^ Missing data between 0% and 47% across groups.

^b^ Missing data between 1% and 68% across groups.

### 2.2 Procedure

The study was conducted as part of a larger, externally funded project about cognitive and emotional mechanisms in paediatric OCD (Cervin et al. 2020). Clinical participants were approached during routine care at the clinic; non‐clinical participants were recruited via local schools. Eligibility for the clinical sample required being clinic‐active during the study period with a principal diagnosis of an anxiety disorder, OCD or a depressive disorder. For the clinical participants, diagnostic status was established by a structured interview conducted by the first author, another licensed clinical psychologist or a trained master's student; diagnostic uncertainties were discussed and resolved in supervision. School‐recruited youth were also interviewed with a structured diagnostic interview and were included only if they did not screen positive. The study was approved by the regional ethics committee (diary number: 2015/663‐3/12; diary number: 2016/92‐12/5). All participants and their legal guardian/s provided written informed consent/assent to participate in the study.

### 2.3 Materials

#### 2.3.1 Diagnostic Status

The Mini International Neuropsychiatric Interview for Children and Adolescents (MINI‐KID) was used to establish the presence or absence of common mental disorders in children and adolescents (Sheehan et al. 2010). The MINI‐KID has shown substantial interrater/test–retest reliability (κ ≈ 0.64–1.00) and good criterion validity versus gold‐standard interviews (specificity ≈ 0.81–1.00; sensitivity ≈ 0.61–1.00) in previous studies (Sheehan et al. 2010).

#### 2.3.2 Self‐Reported Severity of Anxiety, OCD and Depression

All participants completed Swedish language versions of the following self‐report measures of OCD, anxiety and depression: the Obsessive Compulsive Inventory–Child Version (OCI‐CV) (Aspvall et al. 2020; Foa et al. 2010); the Screen for Child Anxiety Related Emotional Disorders–Revised (SCARED‐R) (Birmaher et al. 1999); and the Children's Depression Inventory–Short Version (CDI‐SV) (Allgaier et al. 2012). For the OCI‐CV, we did not include the hoarding items as hoarding is now considered a disorder separate from OCD. For the SCARED‐R, we used the four subscales of generalized, panic, social, and separation anxiety as they correspond to the major anxiety disorders as described in the DSM‐5. Internal consistency (Cronbach's alpha) of all scales was adequate to excellent: OCI‐CV = 0.87; SCARED‐R, generalized = 0.89; SCARED‐R, panic = 0.88; SCARED‐R, social = 0.90; SCARED‐R, separation = 0.77; CDI‐SV = 0.88.

#### 2.3.3 Cognitive Vulnerabilities From the Laval Model

Participants completed four Swedish‐language, self‐report measures of the cognitive vulnerability variables assessed in this study: IU, CA, PBW and NPO. These brief versions have been evaluated for internal reliability, concurrent validity (worry/anxiety) and factor structure consistent with the original scales in non‐referred youth (Fialko et al. 2012) and have demonstrated reliability, convergent validity and sensitivity to change in clinical youth samples across open and randomized trials (Perrin et al. 2019; Wahlund et al. 2022; Wahlund et al. 2020).

IU was assessed with the Brief Intolerance of Uncertainty Scale (Brief‐IUS) (Fialko et al. 2012) comprised of the following five items from the 27‐item IUS for adults (Freeston et al. 1994): *Not knowing what may happen next makes my life horrible*; *I cannot be relaxed if I do not know what will happen tomorrow*; *When I am not sure about something, I cannot get on with it*; *When I am not sure what will happen next, I cannot do things very well*; *Not knowing what may happen next can make me scared or sad*. Each item is rated on a 5‐point scale (1 = *Not at all like me*; 5 = *Completely like me*); higher total scores indicate higher IU.

CA was assessed using the Brief Cognitive Avoidance Questionnaire (Brief‐CAQ) (Fialko et al. 2012), composed of the following five items from the 25‐item CAQ for adults (Sexton and Dugas 2008): *There are things that I would rather not think about*; *I have thoughts that I try to avoid*; *To avoid thinking about things that upset me, I force myself to think about something else*; *I avoid doing anything that reminds me of things I do not want to think about*; *When I have a scary picture in my mind, I say things to myself in my head to replace the picture*. Each item is rated on a 5‐point scale (1 = *Never*; 5 = *Always*); higher total scores indicate higher CA.

PBW was assessed using the Brief Why Worry Scale‐II (Brief‐WW‐II) (Fialko et al. 2012) composed of the following five items from the 25‐item WW‐II for adults (Holowka et al. 2000): *If I worry in advance, I will be less upset if something bad happens*; *Worrying can stop bad things from happening*; *Worrying helps me find a better way to do things*; *Worry helps me to get started on things I must do*; *The fact that I worry shows that I am a good person*. Each item is rated on a 5‐point scale (1 = *Not at all true of me*; 5 = *Completely true of me*); higher total scores indicate higher PBW.

NPO was measured with the Brief Negative Problem Orientation Questionnaire (Brief‐NPOQ) (Perrin et al. 2019) composed of five items drawn from the 12‐item NPOQ for adults (Robichaud and Dugas 2005): *I often doubt my ability to solve problems*; *Often my problems seem unmanageable*; *When I try to solve a problem I often question my own ability*; *I often get the impression that my problems cannot be solved*; *My first reaction to a problem is to question my own ability*. Each item is rated on a 5‐point scale (1 = *Not at all true of me*; 5 = *Completely true of me*); higher scores indicate higher NPO. In clinical samples, the five‐item NPO scale has shown high internal consistency, expected correlations with anxiety, depression, IU, CA and PBW and sensitivity to change in GAD‐focused CBT grounded in the Laval model (Perrin et al. 2019; Wahlund et al. 2022; Wahlund et al. 2020). The five items also overlap with those from a five‐item measure of NPO used in the randomized controlled trial by Holmes et al. (2014), which found the NPO measure to be sensitive to the effects of a GAD‐specific treatment based on the Laval IU model in children (aged 7–12 years).

The psychometric properties of each scale, including internal consistency, were evaluated as part of the present study and are reported under Section 3.

### 2.4 Statistical Analysis

To test the psychometric properties of the four cognitive vulnerability measures in the study, we conducted a confirmatory factor analysis (CFA) using the R library *lavaan*. Because of the ordinal nature of the items, diagonally weighted least squares estimation was used, and robust fit indices were computed. Missing data were low (0.6%), and missingness was handled using pairwise deletion. Good fit was defined as a comparative fit index (CFI) above 0.95, a Tucker‐Lewis fit index (TLI) above 0.95, a root mean square error of approximation (RMSEA) below 0.06 and a standardized root mean square residual (SRMR) below 0.08, while CFI/TLI above 0.90 was defined as adequate fit (Hu and Bentler 1999). A four‐factor model with four correlated first‐order factors was fitted and compared to the model‐data fit of a single‐factor model where a single latent factor explained covariance among all items. Internal consistency (α) for the scales was estimated as part of the CFAs. We evaluated the four scales in a joint CFA model as they are theoretically linked, and a joint model makes it possible to examine whether they capture distinct constructs and how these constructs are associated.

Group differences were tested within a structural equation modelling (SEM) environment where correlations among the dependent variables (i.e., the cognitive measures) were included. Age and sex were accounted for in all group difference analyses. We also tested a model in which associations between age and sex and the cognitive measures were examined. In this model, only scores from the clinical youth were included. Last, we fitted a SEM model where symptom domains (anxiety, OCD, depression) were regressed onto the cognitive measures. This model was conducted with and without accounting for age and sex. For both the CFAs and the SEM model, we used the full sample (i.e., both clinical and non‐clinical youth) to get more variation, to increase statistical power and provide a better representation of the dimensional nature of both the symptoms and the cognitive processes examined (Cuthbert 2014; Fisher et al. 2020). Missing data for the symptom measures used in this model were low (1.1%), and missingness was handled using pairwise deletion. Standardized regression coefficients (βs) were interpreted according to the following criteria: βs below 0.20 were considered to indicate a small effect, βs of 0.20 and above but below 0.50 were considered to indicate a moderate effect and βs of 0.50 and above were considered to indicate a large effect (Acock 2008). An alpha level of 0.05 was used in all analyses. Following Rubin (2021), we did not adjust α across tests because our inferences are based on individual hypotheses (CFA/SEM parameters) rather than a disjunction claim (‘at least one effect’), and exact *p*‐values are reported throughout.

To estimate the average contribution of each cognitive measure to variation in the symptom measures, we ran multivariable linear regression models that included the four cognitive measures as independent variables alongside age and sex and each symptom domain scale as the dependent variable. We first examined the proportion of explained variance in the dependent variable and then carried out a dominance analysis in which all subsets of independent variables were run separately to estimate the average contribution of each independent variable to the variation in the dependent variable. The statistical script is included as a supplement and the data needed to reproduce the results can be retrieved from the corresponding author upon request.

## 3 Results

### 3.1 Fit of the Four‐Factor Cognitive Model

The four‐factor cognitive model that included four correlated first‐order factors corresponding to the scales of IU, CA, PBW and NPO showed good model‐data fit according to all fit indices (CFI = 0.981, TLI = 0.979, RMSEA = 0.060, SRMR = 0.065). The single‐factor model showed poor fit according to all fit indices (CFI = 0.831, TLI = 0.811, RMSEA = 0.178, SRMR = 0.156). In the four‐factor model, the internal consistency (α) for the items of each scale/factor was adequate: IU = 0.91; PBW = 0.71; CA = 0.88; NPO = 0.95. All items loaded statistically significantly onto their proposed factor with all standardized item loadings being above 0.50 except for two of the items of the PBW scale (*Worry helps me to get started on things I must do*, standardized loading = 0.36; *By worrying it shows that I am a good person*, standardized loading = 0.48). We fitted the four‐factor model using only scores from clinical youth and again the model showed good model‐data fit according to all fit indices (CFI = 0.977, TLI = 0.973, RMSEA = 0.060, SRMR = 0.071) and adequate internal consistency across the scales (IU = 0.90; PBW = 0.71; CA = 0.85; NPO = 0.94). The four‐factor model was used in all subsequent analyses.

### 3.2 Group Differences in Relation to Cognitive Vulnerabilities

In a model that accounted for age, sex and correlations among the four cognitive vulnerability measures, the OCD group had higher scores than the non‐clinical group on IU (β = 0.35 [0.21–0.48], *p* < 0.001), CA (β = 0.33 [0.19–0.48], *p* < 0.001) and NPO (β = 0.42 [0.29–0.55], *p* < 0.001), but not on PBW (β = 0.08 [−0.08–0.24], *p* = 0.32). All significant differences were in the moderate range. Similarly, the anxiety group had higher scores than the non‐clinical group on IU (β = 0.46 [0.34–0.59], *p* < 0.001), CA (β = 0.29 [0.14–0.43], *p* < 0.001) and NPO (β = 0.52 [0.40–0.63], *p* < 0.001), but not on PBW (β = −0.03 [−0.20–0.14], *p* = 0.76). All significant differences were in the moderate to large range. No statistically significant differences emerged when comparing the OCD and anxiety disorder groups (all *p*'s > 0.11 and all β's < 0.12). We ran a sensitivity analysis within the combined OCD/anxiety cohort, comparing those with versus without any anxiety disorder (*n* = 123 with an anxiety disorder vs. *n* = 39 without an anxiety disorder; 55% of the OCD group had a co‐occurring anxiety disorder). Scores on the four cognitive vulnerability measures did not differ by anxiety‐disorder status (all *p*'s > 0.21). The group with depression was not examined here due to its small sample size (*n* = 18), but it should be noted that this group had the highest mean scores on NPO and CA among all groups (see Table 1).

### 3.3 Age, Sex, and Cognitive Vulnerabilities

In a model where associations between age, sex and the cognitive vulnerability measures were examined among clinical participants, results indicated that older age was significantly associated with more difficulties with IU (β = 0.19 [0.05–0.33], *p* < 0.01) and PBW (β = 0.29 [0.15–0.42], *p* < 0.001), but not with NPO (β = 0.08 [−0.06–0.22], *p* = 0.26) and CA (β = 0.09 [−0.05–0.24], *p* = 0.21). The significant age associations were in the small to moderate range. Girls reported more difficulties than boys on IU (β = 0.19 [0.05–0.33], *p* < 0.001), CA (β = 0.17 [0.03–0.31], *p* = 0.02) and NPO (β = 0.28 [0.14–0.41], *p* < 0.001), but no significant differences between girls and boys emerged for PBW (β = −0.06 [−0.20–0.09], *p* = 0.45). Significant differences between girls and boys were in the small to moderate range.

### 3.4 Dimensional Associations Between Cognitive Vulnerabilities and Symptoms

**FIGURE 1:**
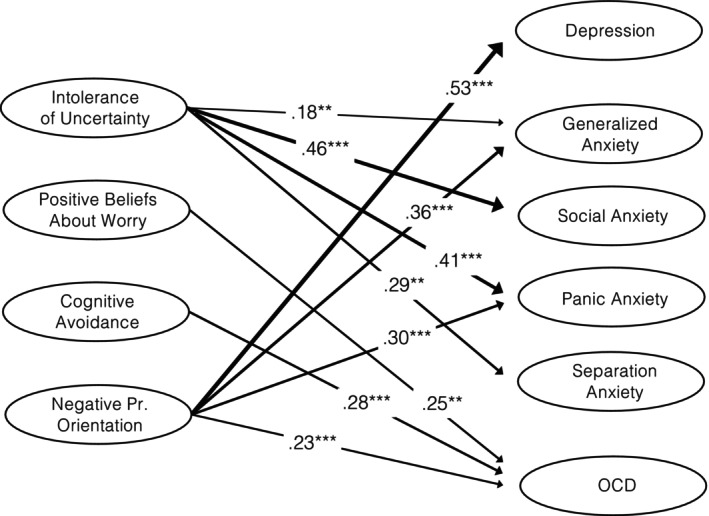
Path model showing the statistically significant associations between cognitive vulnerability measures and symptoms. Standardized regression coefficients are presented. The width of the arrow reflects the strength of the association. * indicates *p* < 0.05. ** indicates *p* < 0.01. *** indicates *p* < 0.001.

The full measurement model for all symptom domains and cognitive vulnerability measures showed adequate model‐data fit (CFI = 0.922, TLI = 0.920, RMSEA = 0.039, SRMR = 0.094). We regressed the symptom variables on cognitive vulnerability variables alongside age and sex. Detailed results are presented in Figure 1. IU was significantly associated with all anxiety factors but not with depression and OCD. CA and PBW were significantly and moderately associated with OCD. NPO was significantly and moderately associated with OCD, generalized anxiety, and panic anxiety and strongly associated with depression. The largest associations emerged between NPO and depression (large effect), IU and social anxiety (moderate to large effect), IU and panic anxiety (moderate effect) and NPO and generalized anxiety (moderate effect).

### 3.5 Average Contribution of the Cognitive Vulnerabilities to Variation in Symptoms

The multiple linear regression models showed that the cognitive vulnerability measures alongside age and sex explained 52% of the variance in generalized anxiety, 51% in depression, 48% in panic anxiety, 29% in separation anxiety, 31% in OCD and 18% in social anxiety. Results from the dominance analyses are presented in Table 2. Overall, IU contributed around 10% to all symptom domains. CA contributed most clearly to OCD. PBW contributed weakly to all symptom domains and NPO contributed substantially to depression, generalized anxiety, and panic anxiety.

**TABLE 2:** Average contribution of each independent variable to variance in each symptom domain according to dominance analysis.

	Depression adjusted *R* ^2^: 51.3%	Generalized anxiety adjusted *R* ^2^: 52.2%	Social anxiety adjusted *R* ^2^: 18.0%	Panic anxiety adjusted *R* ^2^: 47.8%	Separation anxiety adjusted *R* ^2^: 29.1%	OCD adjusted *R* ^2^: 31.8%
Intolerance of uncertainty	10.5%	11.2%	12.5%	17.0%	8.2%	8.1%
Positive beliefs about worry	2.0%	3.7%	0.6%	1.2%	0.8%	6.8%
Cognitive avoidance	4.7%	5.5%	1.1%	5.6%	3.2%	9.8%
Negative problem orientation	24.8%	17.2%	4.1%	16.0%	5.2%	7.0%
Age	6.2%	9.6%	1.2%	4.7%	11.1%	0.9%
Sex	4.3%	6.5%	0.8%	4.8%	2.5%	0.5%

## 4 Discussion

The present study adds to a limited body of research evaluating the relevance of the four cognitive vulnerabilities specified in the Laval Model of excessive worry and GAD (Dugas et al. 1998) to internalizing disorders in youth. First, using CFA, we observed a good model fit and adequate internal consistency of the proposed four‐factor cognitive vulnerability model, suggesting that these constructs are reasonably well defined and relevant in young populations. In this respect, the present study replicates the findings of Fialko et al. (2012) using the same measures in English‐language versions with a large sample of non‐referred youth. Second, the study adds to a small body of literature which finds that these cognitive vulnerabilities are elevated among clinically referred youth, except for PBW. Third, our findings add to a youth literature that has mainly focused, in a univariate fashion, on IU and PBW, by showing that all four cognitive vulnerabilities relate to anxiety, OCD and depression. This is in line with previous research indicating that the cognitive vulnerabilities included in the Laval Model may be relevant across several different internalizing disorders (Akbari et al. 2022; Fergus and Wu 2010; Gentes and Ruscio 2011; Osmanağaoğlu et al. 2018).

When we used a dimensional approach, where cognitive vulnerabilities and major symptom domains were analysed jointly (CFA/SEM), similar results emerged, particularly for IU and NPO, which were associated with several different symptom domains. Notably, in the joint model, IU (originally developed in relation to GAD) was more strongly linked to social anxiety than to GAD. This shows the strength of modelling symptom domains jointly, as such patterns may be obscured in disorder‐specific analyses. At the same time, the joint model also suggests a clearly transdiagnostic role of IU, as it was uniquely related to all anxiety domains. NPO demonstrated significant associations with depression, generalized anxiety, panic and OCD, suggesting an important role for negatively biased interpretations of everyday stressors as ‘threats’ or ‘problems’ for which the individual has limited problem solving ability and/or a low expectancy of a positive outcome (D'Zurilla and Nezu 2010; Kraft et al. 2023). Notably, CA showed its strongest association with OCD, and this may reflect the overlap between the items of the CA scale and cognitive avoidance/neutralizing symptoms of OCD and the prominent role of avoidance in OCD (Cervin 2022). While these results highlight the relevance of cognitive vulnerabilities to internalizing symptoms in youth, the distinct contribution to each domain was limited, indicating that while these vulnerabilities may help explain variance in the severity of the internalizing symptom domains measured here, they do not act alone in doing so, as a substantial proportion of the variance in symptom severity remained unexplained.

Our results also showed that the four cognitive vulnerabilities were more strongly associated with internalizing symptoms of the distress and misery type (e.g., generalized anxiety and depression) (Kotov et al. 2017) than with fear‐based, episodic distress or anxiety, as in OCD or separation anxiety, often triggered by specific situations (Craske and Stein 2016; Foa and McLean 2016). This difference may reflect how distress‐related disorders and symptoms, such as depression and generalized anxiety, involve pervasive, ongoing cognitive patterns (such as chronic worry or a persistent sense of inadequacy) that align more closely with the cognitive vulnerabilities in the Laval model, whereas fear‐based disorders are often characterized by situational avoidance and acute, fluctuating anxiety and distress responses (Stevanovic et al. 2024).

We also found some age and sex differences in relation to the cognitive vulnerabilities. Older participants displayed greater difficulties with IU and PBW, suggesting that as youth age, they may become more sensitive to uncertainty and increasingly view worry as a beneficial strategy. This could reflect developmental changes in cognitive ability and processing, as well as the fact that older youth begin to face more complex life challenges and responsibilities (Crone and Dahl 2012; Steinberg and Morris 2001). Some sex differences were also evident, with girls with internalizing disorders reporting higher levels of IU, CA and NPO compared to boys. This aligns with broader research indicating that women are more likely to exhibit rumination and avoidance as coping strategies in response to stress and have higher rates of depression and GAD (Merikangas et al. 2010).

As noted in the introduction, although the present study did not evaluate treatment effects, CBT programs specifically targeting one or more of the four cognitive vulnerabilities have been found to be effective relative to no treatment for youth with excessive worry, GAD, and significant levels of comorbid anxiety disorders and depression; see also Kendall et al. (2020). It remains unclear whether changes in these cognitive vulnerabilities are necessary for symptom change in these multi‐component CBT programs. A recent randomized controlled trial by Wahlund et al. (2022) found that changes in IU measured weekly with the same IU measure as used in this study did not mediate outcomes in adolescents with excessive worry (the majority of whom had GAD). Given our finding that IU and NPO were linked to multiple symptom domains, we view assessment of these vulnerabilities as clinically informative for case formulation and for selecting/augmenting standard CBT components (e.g., exposure, reducing avoidance and modifying threat appraisals) in youth with anxiety and OCD (Cervin et al. 2024; Kendall et al. 2020).

The results of the present study should be interpreted in the light of some limitations. First, the study included clinically referred youth from a specific mental health clinic in Sweden, which may not fully represent the diversity of youth with internalizing disorders across different cultural and socioeconomic backgrounds. This limits the generalizability of the findings to other populations. Additionally, females were over‐represented (particularly in the anxiety and depression groups). While this pattern is common from mid‐adolescence, sex ratios vary by age, especially in OCD, which may affect generalizability. Moreover, sociodemographic missingness in the non‐clinical group was substantial. Although these variables were not entered into any analyses, we note they are incomplete and should be interpreted with caution. Second, as the study used a cross‐sectional design, it only captures associations at a single time point, making it difficult to infer causality. Longitudinal research is needed to determine if IU, CA, PBW and NPO predict onset, maintenance and changes in internalizing symptoms in youth over time. Third, the group with major depression was small (*n* = 18), limiting statistical power to detect potentially significant associations specific to this group. A larger sample of youth with depression might provide a clearer picture of cognitive vulnerabilities unique to depressive symptoms. However, many youth in the OCD and anxiety disorder groups met criteria for depression (around 20%) and even more had elevated depressive symptoms, making the dimensional models a strength. Last, the reliance on self‐report may introduce biases such as social desirability or inaccurate self‐assessment. While self‐report is an invaluable approach to assess personal, inner phenomena like the cognitive vulnerabilities studied here, combining self‐report with parent‐report and observational and experimental measures would be interesting as the incremental value of each assessment method could be examined.

To summarize, this study highlights the relevance of IU, CA and NPO as cognitive vulnerabilities associated with various internalizing symptoms in youth, while PBW may play a less important role. IU and NPO, in particular, emerge as factors linked to various anxiety, depression and worry symptoms, supporting their potential transdiagnostic roles. These findings suggest that incorporating assessments and targeted interventions for IU and NPO in therapeutic settings may improve treatment efficacy, especially in addressing generalized anxiety, depression and worry in youth. However, further research is needed to fully establish their clinical utility.

## Funding

The corresponding author has received funding from the L.J. Boëthius Foundation, Lindhaga Foundation, the Sven Jerring Foundation and Region Skåne that made data analysis and drafting of the present manuscript possible. The funding sources had no role in study design, data collection, analysis of the data or writing of the report.

## Conflicts of Interest

Dr. Cervin receives research support from the Swedish Research Council for Health, Working Life and Welfare (Forte), the Kavli Foundation, the Kamprad Family Foundation, the Lindhaga Foundation, Stiftelsen Clas Grochinskys Minnesfond, the Crown Princess Lovisa's Association, Region Skåne, Fonden för Psykisk Hälsa and Skåne University Hospital's Foundations and Donations and financial compensation from Springer for editorial work outside of the submitted work. The other authors report no conflicts of interest.

## Data Availability

The data needed to reproduce the results of this study is available from the corresponding author upon request.
